# Enacted Restoration of Selfhood: A Kierkegaardian Perspective on Self-harm Among People with Mental Illness

**DOI:** 10.1007/s11013-026-09975-2

**Published:** 2026-02-15

**Authors:** Alexandra Brandt Ryborg Jønsson, Elizabeth Xiao-An Li, Anne Mia Steno

**Affiliations:** 1 Department of People and Technology, Roskilde University, Roskilde, Denmark; 2 Research Unit for General Practice, UiT The Arctic University of Norway, Tromsø, Norway; 3 Søren Kierkegaard Research Centre, Faculty of Theology, University of Copenhagen, Copenhagen, Denmark; 4 Faculty of education and social sciences, University College Copenhagen, Copenhagen, Denmark

**Keywords:** Self-harm, Mental illness, Selfhood, Kierkegaard, Social psychiatry, Service perspectives

## Abstract

Non-suicidal self-harm, particularly the phenomenon of cutting, is gaining increasing academic attention. However, most existing literature approaches this topic from clinical, cultural, or historical perspectives, often neglecting an in-depth exploration of the lived experiences of individuals engaging in self-harm. Notably, within the context of mental illnesses, self-harm is often viewed merely as a symptomatic behavior and consequently addressed solely through symptomatic treatments. This article draws on data from the first and last authors’ extensive projects focusing on individuals living with mental illnesses in Denmark. Over a period of four years, we closely followed 19 participants (in two different projects) in various aspects of their daily lives, including periods of hospitalization. The article also includes uncensured images of self-harm, provided by participants with their permission to publish. We aim to underscore self-harm not only as the experiential nexus of a complex relationship between the body and the world but also as a mechanism for overcoming the self. Drawing inspiration from Kierkegaard’s reflections on selfhood and despair, we propose the concept of ‘enacted selfhood’ as an analytical framework for comprehending self-harm among individuals living with mental illness.

## Introduction

*‘I can easily remember the first time I self-harmed: It was in 6*^*th*^* grade crafting class when I deliberately cut my wrist with a woodworking plane. Everyone panicked but me: screaming classmates and blood everywhere. I was taken to the emergency room feeling so relieved that I was alive. Like, seeing the blood had proved to me that contrary to how I felt inside, I was still very much alive’* Billie, 29

This article is about self-harm and the role it may play in a person’s sense of selfhood. As the above quote from Billie illustrates, self-harm reflects a complex and vexed relationship between body and world, one whose meaning depends on the perspective of the observer. In our research on living with mental illness, self-harm unexpectedly became the locus of conversations, relations and emotional expressions. It appeared as acts of ‘freedom,’ as shared secrets, and ways to establish a feeling of intimacy and trust between us. What our interlocutors showed and told us challenges conventional understandings of self-harm, revealing it as a way of “finding a path back to oneself”, as Kimmie, a woman in her late 20s said. In understanding self-harm through the lens of restoring selfhood, Danish philosopher Søren Kierkegaard’s (1813-1855) conceptualization of existence is well-suited, since Kierkegaard emphasizes how existence is both given and poses a challenge to each individual (Kierkegaard 1848, 1980). This framework adds to current understandings of self-harm as something that may also be a conscious act symbolizing the despair of feeling disconnected from one’s true self or longing to be otherwise.

The act of self-harm; deliberately inflicting pain or injury upon one’s body, is often seen among people living with mental illnesses, in particular, but not limited to, bipolar disorders (Nitkowski & Petermann, 2011; Weintraub et al., 2017) and schizophrenic disorders (Jakhar et al., 2017; Nordentoft, 2007). It takes many shapes; cutting, eating disorders^1^, pouring acid drain over one’s body, attempting to remove genitals, etc. and is commonly divided into non-suicidal self-harm and suicidal attempts.

Medical literature describes self-harm as symptomatic behaviors closely tied to psychiatric, psychological, familial, social, and cultural factors (Hawton et al., 2012). Within this literature, the primary focus lies on prevention, treatment (Morken et al., 2019), and the identification of high-risk individuals (Rasmussen et al., 2016). In addition, a substantial body of research has documented the relationship between self-harm and trauma-related disorders, including PTSD and complex PTSD. Studies demonstrate that experiences of childhood abuse, neglect, and other forms of interpersonal trauma can be central to the onset and persistence of self-harming practices (Briere & Gil, 1998; Ford & Gomez, 2015). This connection highlights that self-harm cannot be understood solely through diagnostic categories such as bipolar or schizophrenia, but must also be situated in relation to the enduring effects of traumatic experiences, though not necessarily as psychiatric entities, but as lived disruptions that shape subjectivity and self-relation. It is often argued that mental disorders such as schizophrenia and bipolar disorder profoundly shape experiences of selfhood (Sass & Parnas, 2003). Our aim is not to question the validity of diagnoses or traumas as such, but to explore how these categories intersect with lived experience. While psychiatric diagnoses are themselves uncertain and socially negotiated categories (Jutel, 2009) they nonetheless shape how people understand and articulate their suffering. We therefore approach our interlocutors first and foremost as persons rather than as patients, while acknowledging that their mental illness is a powerful social and experiential reality in their lives.

In the social sciences, self-harm is also seen as easing emotional worries (Gardner, 2013) or a way of communicating using the body as language (Motz, 2009). The latter aptly argues that viewing self-harm as subjective leaves out important aspects: promoting self-harm as something individual rather than social, private rather than relational (Steggals et al., 2020). The diverse meanings ascribed to self-harm in both medical and social science literature introduce a layer of complexity. Whether viewed as a method for emotional regulation, self-punishment, interpersonal manipulation, negotiation of care, coping mechanism, attention-seeking, emotional expression, or communication of distress, these perspectives contribute to a structured ontology of self-harm that is subject to rational understanding (Adler & Adler, 2011; Harris, 2000). This rationalization often leads to conflicting viewpoints between individuals who engage in self-harm and healthcare professionals (Hadfield et al., 2009; McHale & Felton, 2010). This calls for closer attention to the illness narratives of people who self-harm (see Chandler, 2014), and we further argue that it is important also to understand the embodied lived experience that underpins the processes of self-harm, not least when self-harm is related to mental illnesses. As pointed out by Csordas and Jenkins (2018), most of the literature on self-harm, including self-cutting, is written from clinical or psychometric standpoints, leaving a gap of ethnographic knowledge on self-harm as lived experience.

In this paper, we draw on data from two studies of adolescents and young adults living with mental illness in Denmark. All our interlocutors are or have been self-harming. Some also have a record of suicidal attempts, but in this article we focus on non-suicidal self-harm, because these types of self-harm have different drivers, as we learned in conversations with interlocutors. Our focus on self-harm among people who have been clinically defined and diagnosed does not interfere with our ethnographic stance that self-harm can be a symptom of a mental illness and simultaneously an expression of a vexed relation between body and world. Rather, we emphasize the relation between psychiatry and social suffering, as pointed out by Kleinman (Kleinman, 2012, p.183).

The aim of this paper is to contribute to the literature on self-harm suggesting that self-harm may, for some, operate as a means of restoring selfhood through the application of a Kierkegaardian perspective. This interpretation does not supplant other explanatory frameworks but supplements them by attending to the existential dimensions of embodied suffering. Following this, we explore the complicated mixture of coercion and emancipatory actions performed in self-harm among people living with mental illnesses in Denmark.

## Methods and Ethnographic Context: Living with Mental Illnesses in Demark

Mental illness remains a contested term (Good, 1997; Szasz, 1961). Following Luhrmann’s call to acknowledge the diverse and culturally situated forms that mental illness can take and the profound disruptions and suffering it often entails (Luhrmann, 2016, p. 12), we view mental illness as both socially constituted and profoundly real in its effects. We focus here on the lived experiences of individuals diagnosed with schizophrenic or bipolar disorders, which typically require ongoing medical treatment and often leave lasting traces of struggle, vulnerability, and social exclusion in the lives of those affected. Indeed, struggle is deeply woven into the everyday lives of our interlocutors. Living with voices, managing symptoms, treatments, psychosis, stigma, and discrimination are part of their daily reality; an experience all too common among those living with mental illness (Jenkins, 2010, p. 2). To those who self-harm, a mutilated body bears silent witness to this struggle. Hence, we do not wish to fall prey to what Luhrman has named the “romanticizing” argument; that mental illness, madness, is only a cultural construct, even though symptoms are to be interpreted in a cultural context (Luhrmann, 2016, 10ff). Likewise, there is a prominent understanding in the public consciousness that mental illness is conducive to creativity, productivity, or other talents, for which however there is very little evidence (Martin, 2007). The fact that many people who have been considered geniuses were subject to harsh life experiences, self-harmed or experienced mental instability does not mean that clinical, debilitating mental illness was a contributing factor to their eminence (Ibid; Kaufman & Sternberg 2010, 381-383). Among our interlocutors, mental illness is a destructive and intractable disease (Luhrmann, 2016), which they would have rather been without.

During 2016-2021, Anne Mia conducted ethnographic fieldwork at four housing facilities for people with mental illnesses for the research projects “Social pedagogical work with relationships among young adults with mental disorders” and “Legitimacy and agency”. These housing facilities are all municipal pedagogical services, which in their own words: “offer housing for persons who, due to significant physical or mental disability or special social problems, have a need for it”. The offer is temporary, which means that the goal for individual residents is to move on to more independent forms of housing after a shorter or longer period, usually a few years. The service users have their own private room, and there are common areas such as a kitchen and TV room. The staff strives to create a homely atmosphere and informal interaction with the residents. Anne Mia spent time with the interlocutors, gamed with them, smoked with them, went to social gatherings and family visits with them. In addition, she conducted recurring in-depth qualitative interviews with 12 interlocutors, 3 times with each individual.

As part of the SOFIA trial (see Rozing et al., 2021) Alexandra has done ethnographic fieldwork among people diagnosed with bipolar or schizophrenic disorders since 2018. Contact to interlocutors was established through either an interlocutor’s General Practitioner or Psychiatrist asking if they were willing to be interviewed. Twenty-two people were interviewed about their life-story and health practices, and eight of them agreed to be part of a longer fieldwork period including continuous visits, conversations and interviews with Alexandra. Since the official ending of the project in 2020, three people have remained as interlocutors to be followed in more longitudinal ethnographic research with conversations and visits a couple of times per year. Visiting interlocutors in their homes (assisted living or independently) and taking part in everyday life chores and social life has shown life with mental illness to be fluctuating, challenging and yet also rich and meaningful.

Both studies were conducted in accordance with The Danish Code of Conduct for Research Integrity and AAA’s ethical guidelines. Names and places have been anonymized and participants have given their informed consent. A cornerstone in research involving human beings, informed consent is intended to safeguard voluntariness but has been criticized for disregarding research that involves individuals who may experience intellectual, cultural or other barriers in regards to questioning authority (Høyer, 2009). In our setting, it was important to ensure that participants understood that their participation, or withdrawal, and what they would tell, would not be shared with any health care professionals or influence their treatment and recovery plans. We conducted most of our research in private homes or in spaces of the housing facilities that were considered ‘safe’ from eavesdropping. We initiated contact with interlocutors explaining that the overall aim was to understand everyday life with severe mental illness. We also stressed that they would not be identifiable in the results and therefore emphasized that even though their voice is important it would be perfectly fine to withdraw at any time without having to provide any explanation for such a withdrawal.

All interviews were audio recorded and transcribed, and the interlocutors were offered to read through the transcripts. Fieldwork notes would be written during fieldwork or as soon after as possible and were likewise available to interlocutors. Billie preferred to record her own story on her phone and send it to Alexandra and then go over the transcript to discuss it afterward which would prompt questions from Alexandra. However, formal interviews with Billie were also conducted. The photos included in this paper are privately taken by the interlocutors themselves and shared with their permission. They were not produced as part of this research project, but were shared in conversations about self-harm, as a way for interlocutors to illustrate and communicate how their cutting appeared and what it meant to them.

All our interlocutors live with functional impairments due to their mental illnesses. They either receive early pension or are in job training of some kind, usually for a limited number of hours a day, and receive additional financial aid. Whether living in assisted housing facilities or independently our interlocutors receive social service support to help functioning of the individual, including visits twice a week from a coach, assistance with grocery shopping or attending doctor’s appointments.

Treating self-harm is in Denmark conducted by health care services divided into regional mental health centers providing inpatient units, outpatient clinics, community mental health services, outreach services and specialized treatment (Davidsen et al., 2020). All health services are free to citizens without co-pay, as they are publicly financed and likewise all prescription drugs are subsidized (Vrangbæk, 2016). The most common treatment strategy is cognitive therapy (Erlangsen et al., 2015). Acute self-harm and suicidal attempts are treated at emergency clinics. If suicidal, patients are usually hospitalized until they no longer are perceived to constitute a threat to themselves or others. Emergency clinics can be accessed directly, but mental health treatments require a referral from a general practitioner (Davidsen et al., 2020).

**Fig. 1:**
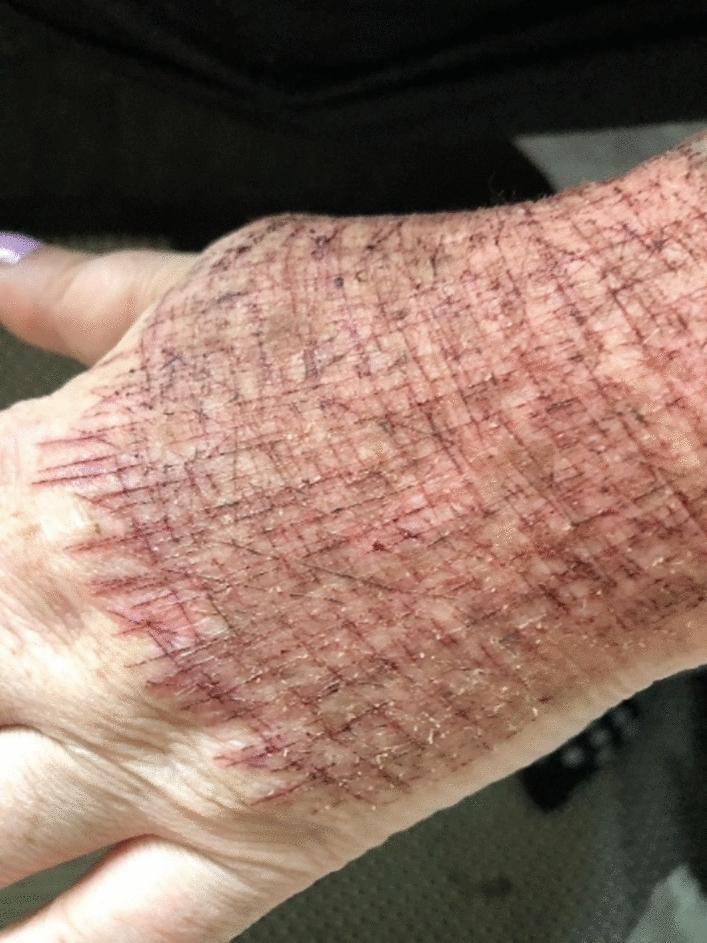
Picture of Kimmie’s left hand

Regardless of setting, self-harm is viewed through the professional lens as something pathological; something to be cured or eliminated. Many of our interlocutors share this desire to reduce self-harming behaviors and actively work toward it. Kimmie, for example, has managed to limit her cutting to three times a day, down from six, always in the same place on her hand. See Fig. 1.

The significance of the image lies not in its graphic nature, but in the fact that Kimmie shared it only after trust had been established between us. She always wears gloves, and revealing her scars was a gesture of the relationship we had built, as is permitting us to use this figure for academic publication. Once trust was established, Kimmie told Alexandra that although she wished to stop self-harming, the wounds also served as a reminder of who she was: a survivor, someone who could endure pain.

Ethnography always calls for collaborations between anthropologist and interlocutors. In this article, we draw on various narratives and stories of interlocutors, but Billie has played a particular role: during fieldwork she began engaging in analytical perspectives on her own story, and she is the one who coined self-harm actions as a ‘restoration of selfhood’ [*en måde at blive sig selv igen*, literally: “a way to become yourself again”]. This is why her narratives play a prominent role in the empirical data of this paper. Stylistically, we have chosen to include her full narrative as a subsection, to underscore the role of childhood trauma in her experience of losing her sense of self. For personal reasons, Billie wishes to remain anonymous and has declined authorship. In light of Billie’s articulation of selfhood, Kierkegaard scholar Elizabeth, has contributed the theoretical perspective of Kierkegaard on which the further interpretation draws.

## Billie: My Story (as told to Alexandra)

I was born in a suburb south of Copenhagen on a cloudy December day in 1993. Shortly after my 10^th^ birthday, my abusive father left never to be seen again, and I grew into adolescence with an alcoholic mother in a low-income area with high crime rates and local gangs. On instable grounds, the rough environment of the gangs, and the young men who addressed me as “princess” immediately attracted me. Within months, I was lured into a basement and raped by an old man: my ‘friends’ from the gang had just told me to sit in a room and wait, and then he entered and forced himself upon me. But after the rape he gave me a brand-new Louis-Vuitton (designer) bag. I was deeply traumatized by the rape, but I was also drawn to the promise of expensive gifts, I could have things I had only dreamed of before. So, I began selling sex, and the gang members started finding clients for me. I guess that to bear the pain and trauma I started abusing illegal drugs, and by the time I began to self-harm in 6^th^ grade, I was bringing half a liter of vodka to school, most of the time high on coke or marijuana. I had lost all tactile senses of my body, and when I tried H (heroin) I was unable to even feel the syringe breaking through my skin, and got really confused and scared because I did not know whether I was alive or not. That was when I came up with cutting, because I figured, that if I could see my own blood, it was a sign that I was not dead.

To me, this was also the beginning of living with mental illness. At the age of 14, I was placed against my will in a state institution for troubled youths, and I began experiencing psychosis. I was diagnosed with bipolar disorders and paranoid schizophrenia, and by the age of 19 I was forcibly admitted to the psychiatric ward where I spent most of the next 8 years. Here, my self-harm increased, and I began having recurring suicidal attempts, which caused the forced admittance.

(*Can you recollect one of these incidences?)*

Yes, a couple of years ago, during Covid, they decided to release me from the psychiatric ward, and that’s when my head went crazy. Going home to an empty apartment freaked me out. I’m telling it as it is, I just panicked, I was like, “I can’t be alone without anyone to look me in the eyes”. I asked to be hospitalized again, but the head-psychiatrist refused because of these new, I call them their nazi-rules, which stated that I couldn’t be hospitalized because I had jumped out of a 2nd floor window to avoid being forcibly restrained. So, home I went, and the minute I walked in, I called my contact person at the municipality yelling that something is wrong. She must have sensed some sort of panic in my voice because then she calls for emergency back-up, and an ambulance and the police arrive. That’s when my head just goes black, I need to feel something, and I run into the kitchen, grab a knife and stick it in my heart (…) the police storms the apartment, dogs and policemen everywhere (laughing) I’m on the floor crying in pain because of the pepper spray they used to get control of me, and I’m just yelling “I’m ok, I’m alive, go help someone who’s really in need”.

(*You always seem so positive, even when you tell these stories, is that a strategy to overcome?)*

**Fig. 2:**
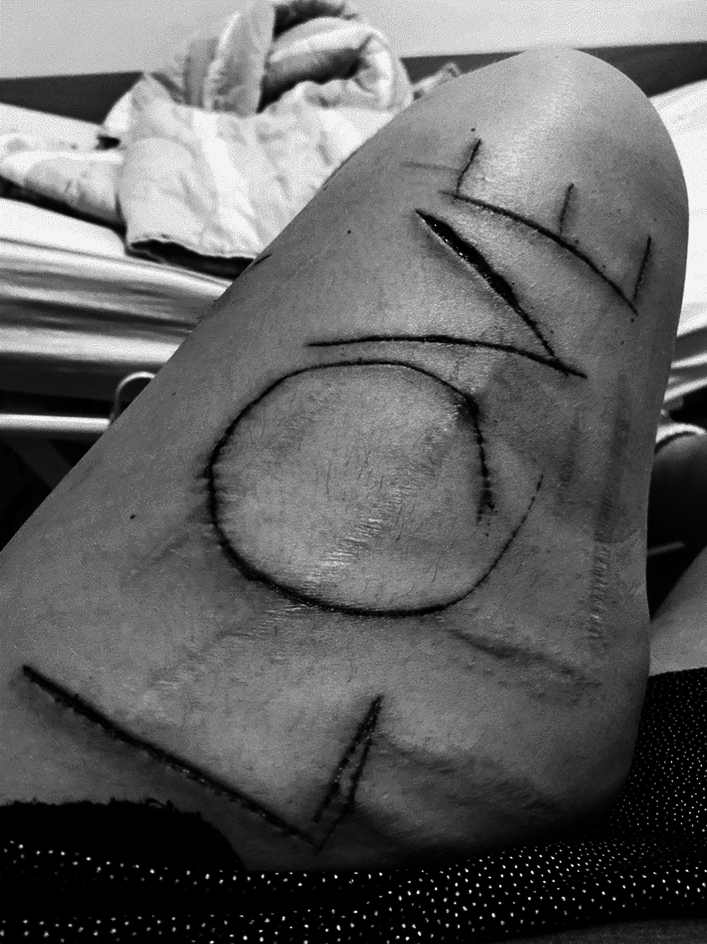
Picture of Billie’s Upper leg. Photocredit: Billie

**Fig. 3:**
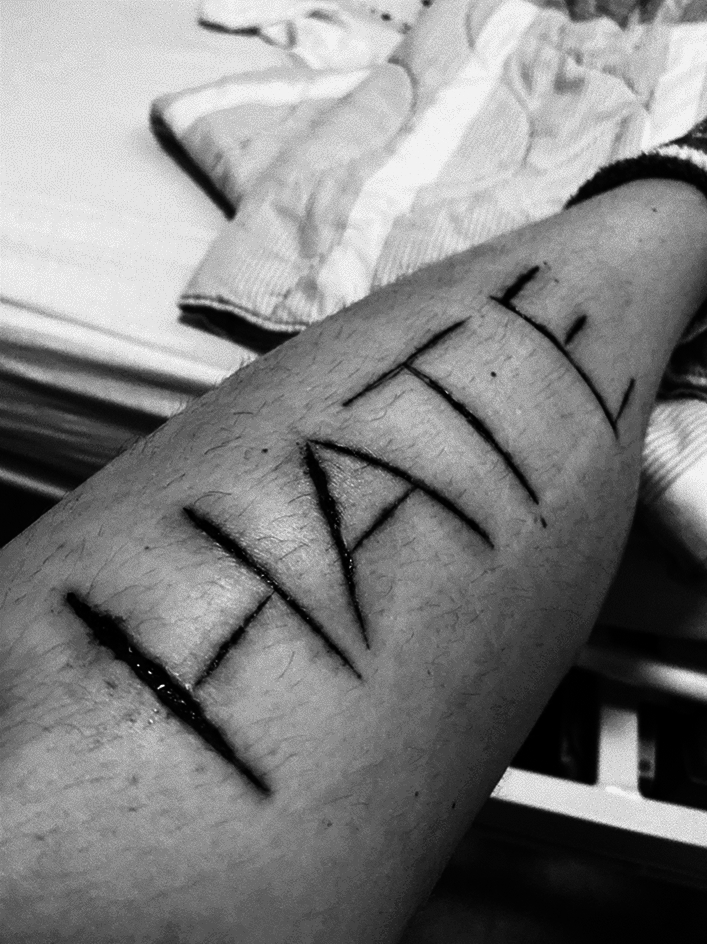
Picture of Billie’s lower leg. Photocredit: Billie

Well, as I see it you only have one life, so you must make the best of it, but it’s been really hard. Apart from my substance abuse and abusing my body sexually, since I did not take any joy in the sex I sold, I started cutting myself with razor blades, inscribing words or making wounds just to see the blood run. I took these figures immediately after one of my admittances to the psychiatric ward. I was very confused and began questioning my own existence. I was like, I always am, asking myself: do I exist? So, I needed to cut and see my own blood flowing as proof. The particular wordings here symbolize my feeling of being overwhelmed by the institution and a need to inscribe something that defined me as a person; ‘love’ for my personality and ‘hate’ for what I experienced from others (see Figs. 2 and 3)

Blood became sacred to me, a symbol of life. If I was unable to get my hands on razorblades, for instance when hospitalized, I would instead bang my hand against the wall or floor in the quest for blood. Broken bones did not make me stop, because all my tactile senses were being put out of play. Not until I could see blood, did I know for sure that I was alive. The same mechanisms were present during my eating disorder, not least when I had my bulimic attacks; I did not throw up only to lose weight but to see the blood coming from my throat and ulcer.

The eating disorder is the one thing that can still bring me down. I am proud to say that I have not self-harmed since April 2^nd^ 2022, but staying away from sharp objects is much easier than recovering from an eating disorder. My self-harm has never been a cry for help, it was about selfhood, to get a glimpse of Billie. During my teen years when everything was so fucked up, I came up with an alter ego; Pink. But with the self-harm and the abuse and everything, Pink had taken over my identity. I was no longer in my body, it was Pink who was acting out, experiencing all of this. But when I saw the blood, I knew that Billie was still somewhere deep inside me. My complicated relation with food is a reminder that Pink is not completely gone.

## Self-harm in a Kierkegaardian Perspective

It is an unfitting word, ‘self-harm’, because it really has nothing to do with a wish to harm yourself. Also, it is not something you ‘do’ in isolation, it is more like a reaction, something necessary, that you *have* to do, it’s who you are (Sidsel).

Regrettably, self-harm has evolved into a widely recognized response to human suffering, often stereotypically associated with demographic groups like teenage girls or subcultures such as “Emo” or “Goth” (Csordas & Jenkins, 2018, 206). These subcultures tend to romanticize and foster self-harm, particularly cutting, as a cultural practice (Ibid, Young et al., 2014; Zdanow & Wright, 2013). This prevailing perspective emphasizes a dominant model that frames self-harm as an individual ‘crisis’; a coping mechanism for distress and emotional pain.

Inspired by Csordas and Jenkins’ (2018) insights on adolescent psychiatric inpatients in the American Southwest, we add to the psychological anthropological understanding of self-harm by using the work of Kierkegaard in this analysis. Following Csordas and Jenkins, seeing self-harm as “*a crisis of agency enacted on the terrain of their* [people doing self-harm’s] *own bodies*” (Csordas & Jenkins, 2018, 208), it is precisely the enactment that should be at the core of our understanding. According to Billie and as her story shows, her various ways of harming her body is not a cultural phenomenon, nor a cry for help or attention, but an intricate way of dealing with and relating to her own senses and emotions. Her body is making her feel dead, numb and incapable of sensing pain, yet the body also becomes the literal marker of whether she is alive. Through this perspective, self-harm is also a form of enactment of selfhood. Here, our use of selfhood encompasses the totality of an individual’s experience, thoughts, feelings, and unique characteristics that contribute to their sense of being and personhood. Selfhood thus involves the awareness and recognition of one’s existence, relating to oneself, and the capacity to distinguish oneself from others.

For this reason, Kierkegaard’s existential account of the relational self offers a particularly useful theoretical lens through which to shed light on Billie’s (and others’) complex, ambiguous, and even paradoxical experiences with self-harm. Kierkegaard’s definition that “the self is a relation that relates itself to itself” (1980, p.13) can appear circular or opaque. To clarify: selfhood is not identical to the empirical self, i.e., the collection of experiences, feelings, and bodily states, but refers to the process by which the self turns toward and interprets itself. In this account, the self is both the one who relates and that which is related to; it is simultaneously subject and object. This reflexive relation is what allows a person to experience herself as a self at all. Crucially, this relation is mediated through embodied existence: one’s physical givenness, affective states, and lived experiences form the material through which the self-negotiates possibility and necessity. In this way, selfhood can be said to arise not abstractly but through concrete acts such as self-harm, which, paradoxically, are aimed at a restoration of one’s relation to oneself when such relation has been fractured or obscured.

Before discussing an account of this framework, it is however important to point out that the Kierkegaardian relational self has already been used in health studies to analyze how patients with chronic illnesses struggle to relate to themselves in their new life-situations (Andersen et al., 2021). Specifically in relation to mental illness, Kierkegaard’s ideas have informed spiritual psychotherapy for people with depression (Benning, 2023). Our use of Kierkegaard’s notions of selfhood and despair as a theoretical framework for understanding self-harm as an enacted restoration of selfhood, is moreover inspired by Jeanette Knox’s analysis of cancer patient narratives through a Kierkegaardian theory of the self (Knox, 2019, 2022).

Adding to Kierkegaard, we suggest focusing on the embodied experience of self-harm. As noted by Lester, this includes the perspective of the actor who embodies and responds to cultural systems of meaning (Lester 2012, 727 in Csordas & Jenkins, 2018). Lester suggests viewing cutting as a decontextualized and privatized social ritual, which, in its transformative move between self-harm and repair, may parallel collective initiation rituals (Lester 2012 in Csordas & Jenkins, 2018, 23). Emphasizing the self-harming person as a cultural agent enables new understanding in connection with a phenomenological approach. In an article on self-harm in the context of urban homelessness, Burraway uses critical phenomenological anthropology and psychoanalytic theory to “re-conceptualize self-harm as a mode of being-in-the-world” (Burraway, 2021, 305). This phenomenological approach is used to read self-harm as “a moment-of-the-flesh where being, pain, relief, repair, emotionality, and worldly attunement converge, an ontological and ethical response to the conditions of co-existence” (Burraway, 2021, 305). Hence, it can be argued that self-harm is an attempt at establishing selfhood as well as a “meaningful sensory perception in temporal context and within particular cultural, social, and interpersonal settings and subjectivity as the more or less enduring structure of experience” (Csordas & Jenkins, 2018, 208). Here, enacted restoration of selfhood, is not necessarily about regaining a prior, intact self, but more of a restoration as the re-establishment of relation to oneself after that relation has been fractured by despair. Billie’s description of seeing her own blood illustrates this: it does not return her to a former version of herself, but rather grounds her in existence, providing a tactile certainty that she is alive and that herself still persists. This adds significance to drawing on Kierkegaard’s philosophy of existence for analyzing the depiction of self-harm in Billie’s story in which the shedding of blood becomes a symbolic as well as embodied restoration of selfhood.

### A Kierkegaardian Framework of Selfhood and Despair

In philosophy, psychology and anthropology selfhood is a recurring theme which has led to many different interpretations (Nagel, 1986; Ricoeur, 1990; Taylor, 1989; Zahavi, 2025). Kierkegaard’s existential account of selfhood remains a highly influential approach to this concept, which nevertheless remains contested and discussed in Kierkegaard scholarship (Damgaard, 2017; Grøn, 1997; Jothen, 2014; Rosfort, 2022; Stokes, 2015; Taylor, 1980; van Stee, 2022). It should be noted that Kierkegaard’s writings, often published under pseudonyms, are difficult, multifaceted and open to interpretation.

Kierkegaard’s theory of selfhood can be contextualized within his particular and two-sided account of human existence. Kierkegaard views existence as a dual dynamic: we are already existing as ourselves, but we also have to become or determine the particular or individual selves that we are, as existence is a constant state of becoming (Kierkegaard, 1992; Knox 2022, p. 2902.). In other words, our existence is simultaneously given to us but also presents us with the task or problem of determining ourselves as the selves we are (see also Grøn, 2022, 29-33). This task is made clear in Kierkegaard’s famous definition of the self as: “a relation that relates itself to itself, or is the relation’s relating itself to itself in the relation… A human being is a synthesis of the infinite and the finite, of the temporal and the eternal, of freedom and necessity, in short, a synthesis. A synthesis is a relation between two” (Kierkegaard, 1980, 13). Thus, a self is firstly understood as a dynamic self-relation, because it is tasked with relating or synthesizing the dual sides of its existential condition (that it is both already in the embodiment, finitude, temporality and necessity of existence, while at the same time having the freedom and possibility to determine itself in seemingly infinite ways). For a person to truly become themselves, they must be able to reflect on who they are and to relate to the different parts that make them up, such as their body, mind, and feelings. This reflective relationship with oneself is not something that others can give or teach, rather it’s something each person must take up on their own. Becoming oneself, in this sense, is a deeply personal task that requires self-awareness and inward engagement rather than external guidance.

A special feature of Kierkegaard’s concept of selfhood is that despair plays a crucial role in the establishing of our selves. Because the self is a synthesis, a relation that relates itself to itself, Kierkegaard points out that *mis*relation, or failing the task of becoming a self, is not only possible, but also an inevitable experience of being a human. And such misrelation is despair. His analysis of existence includes acknowledging the many forms of despair that one can find oneself in, which in different ways express such misrelation to one’s self either by not wanting to be oneself or defiantly wanting to be oneself. Despair, however, stands in our reading not as an unwanted obstacle but a pivotal part of the subjects’ journey toward selfhood: “You must go through the despair of the self to the self (…) the self must be broken in order to become itself” (Kierkegaard [1848, *Original in Danish*]1980: 65).

### Self-harm, Selfhood and Despair

As such, Kierkegaard provides a theoretical grounding for understanding how self-harm becomes an enactment or a conscious act symbolizing or expressing the despair a person is in when experiencing themselves as something they are not or wanting to be something other than they are.

Micki and I were strolling along the small lake in their neighborhood. My intention was to ask about the recently concluded art-classes that had been a part of their therapy, but instead Micki showed me a new and rather severe looking scar from acid on their left underarm, saying: “the ER nurse got really pissed with me, she started yelling and screaming that she should not be dealing with this, I did this myself, so other people deserved her help more”. I looked at Micki with shock, my naïve notion of ER services from the perspective of a protected privileged life could not grasp the hostility of such a statement. Micki just shrugged and remarked that this was a common experience for self-harmers when seeking out help for self-induced wounds. The shared intimacy dared me to ask them; *why do you actually self-harm?* It was a risk to ask, I knew Micki was sick and tired of being assigned intentionality and responsibility for self-harm, but the moment seemed right. “It’s not just one thing”, Micki easily replied, “it’s more…everything. Like it’s something that I do with and to my body but it’s also something that I am. And I am that because I am different from you, you know, if I were alone in the world I don’t think I would self-harm”. Perhaps replying to my perplexed grimace, they laughed and continued “*no, no, I’m not saying it’s anybody’s fault, I don’t know. I feel like connected to the world in the moment, like in Avatar* (a fictional movie), *connected to everything around me. I am truly, 100 percent alive when I do this. And then it hurts like shit* (laughing)”

Perhaps we can better understand Micki’s self-harm in light of the Kierkegaardian account of the human self as a composite being made up of heterogenous aspects. As noted above, Kierkegaard’s account states that the self is a synthesis of the psychical and physical, temporality and eternity, finitude and infinitude, and necessity and possibility (or freedom). These aspects must be related in the endeavor of establishing selfhood, yet, when one realm becomes dominant it creates a misrelation that manifests as despair (Kierkegaard, 1980). If the subject cannot find congruence between the heterogeneous aspects within their own existence it creates misrelation and despair. In modern life, we may tend to regard despair as a misfortune, but in a Kierkegaardian lens, despair is an opportunity for a new self to emerge and thus makes it possible to re-establish selfhood. For Kierkegaard, misrelation or despair constitutes an almost necessary step in moving beyond the negative toward the positive, that is, the self. The self must in some sense “break” to be re-established. At the same time, Kierkegaard develops a paradoxical idea in which the positive reveals itself through the negative: despair becomes a kind of negative relief, showing what must not be done to become a self. Importantly, Kierkegaard does not set out a normative program or prescribe what one ought to do, since becoming a self is an entirely individual task. Instead, he points to how we go astray, what we do wrongly, so that each may consider how to undertake their own task of selfhood. Still, there are likely fundamental differences in the forms of despair that we as subjects experience, and Kierkegaard distinguishes between the misrelation that can range between two forms of despair: the despair of not being willing to be oneself to being in the despair of assertively and defiantly willing to be oneself (Kierkegaard, 1980:13), just as he points out, that one’s consciousness of despair can vary from minimal to maximal. While being in the despair of not being willing to be oneself is an abstract and hard-to-grasp figure, it should be understood as the subject trying to get rid of any ‘self’ opposed by outside factors (see for example Knox 2022:2903-4). Billie explains this as feeling alienated from herself when she was first described by her therapist as a victim: “I felt like I was being belittled, I denied the label and as I remember I walked out on the therapist. But later, I started to embrace this ‘victim’ thing, I began seeing this as my new self, my old one had been broken by trauma, and this new one was not any lesser because of that”. The other end of the scale, the despair of wanting to be oneself in defiance, expresses a desire to solely invent oneself, which is also, to Kierkegaard, a way of losing oneself and not a path to freedom. This dual orientation of despair and its connection to establishing a self can also shed a light on our interlocutors’ accounts of self-harm.

To Le, self-harming was both a way to try to escape herself and the identity connected to a childhood marked by abuse and neglect, but at the same time also a way to find herself within that identity. It may seem counterintuitive, but Le can easily translate this into her lived experience: “When I pull out my hair, I know it’s stupid, but the hair (which is red and curly) has always been a part of me, and therefore also a part of all the crap I’ve been through.” Still, Le’s self-harm is also a path to losing herself, because as she says, “But then sometimes I also feel like if I don’t have my red hair, then who am I?” Here, Le recognizes herself when she is told and reassured by others that she does, in fact, exist, when people recognize her because of her hair. But she cannot fully grasp that this is her, and she feels a desperate need to invent a way of becoming a self that she, too, can recognize. This can be understood as a striving to balance the ever-present tension that Kierkegaard identifies between necessity and possibility.

### Embodiment and the Enactment of Selfhood

While Kierkegaard provides a framework for grasping selfhood as a reflexive relation to oneself, anthropological theories of embodiment remind us that this relation is always mediated through bodily practices and experiences. Selfhood is not an inner essence that is simply expressed through the body; rather, it is constituted through embodied acts that continually reshape subjectivity. Here, Elaine Scarry (1985) has shown that pain can “unmake the world,” dismantling the coherence of language and subjectivity. For many of our interlocutors, however, this unmaking had already occurred through trauma, psychiatric institutionalization, or the numbing effects of medication and despair. In this context, self-harm paradoxically uses pain to make the body perceptible again, interrupting dissociation or emotional overwhelm and grounding the person in their own living embodiment. Ariel Glucklich (2001) similarly emphasizes that pain can have constructive dimensions: it may alter consciousness, reconfigure relations to the body, and even become a source of meaning. Thomas Csordas (1990) proposes that embodiment is not a thing we “have” but the existential ground of culture and selfhood. These perspectives allow us to see acts of self-harm not as expressions of an inner psychological state but as embodied practices that constitute the very possibility of relating to oneself. Rebecca Seligman’s work on Candomblé mediumship further illustrates this point: in ritual possession, bodily acts and altered states do not merely reflect but actively transform the self, creating new ways of being in the world (Seligman 2014). We argue that self-harm dismantles and rebuilds selfhood through embodied practice. To our interlocutors, embodiment is central: selfhood is restored not through abstract reflection but through bodily enactment. Self-harm becomes the medium through which despair is both materialized and momentarily transformed, allowing interlocutors to re-enter a relation with themselves. In this perspective, “enacted restoration of selfhood” becomes an embodied practice through which fractured self-relations are temporarily reconfigured and renewed.

## Establishing Selfhood Through Self-harm

Kierkegaard’s existential account of selfhood, especially the connection between body and selfhood, also translate well to the experiences of Lyng. Lyng, a non-binary person in their 20s, is diagnosed with paranoid schizophrenia, eating disorders and has a history of severe self-harm, including cutting. Yet, the label of self-harm is not always suitable, Lyng explains. Sometimes, it is a matter of pure existence. Being AFAB (assigned female at birth), Lyng felt wretched when they entered puberty, and the physical signifiers of gender, in particular female breasts, caused them to have suicidal thoughts. It can be argued, that Lyng experienced a misrelation in not wanting to be the self that outside, here genetic, factors had assigned. When they tried to cut off their breasts, therapists named it self-harm, while Lyng says: “It was the only way I could go on living”. It can be considered an act of despair that nevertheless aimed at creating balance within the self-relation and constant becoming in selfhood.

Several of our interlocutors described self-harming as their only way to “feel something.” It is important, however, to clarify what kind of “feeling” is meant here. For some, as Lyng’s story illustrates, physical sensation was sought as a way of grounding themselves in their bodies: cuts, burns, or blood offered tactile proof of existence when numbness dominated. For others, physical pain functioned as a way of numbing or displacing emotional pain, creating relief from overwhelming feelings of despair, shame, or panic. In this sense, physical and emotional registers of “feeling” are paradoxically intertwined: one can seek pain both to awaken the body and to quiet the emotions. Our argument is not to equate “feeling something” with “becoming a self.” Rather, we suggest that self-harm creates a temporary condition in which embodied sensation allows the person to re-establish relation to themselves whether by awakening what feels absent or by dampening what feels unbearable. In Kierkegaardian terms, this moment represents *not* the resolution of despair, but a temporary recalibration of the misrelation between possibility and necessity. However, self-harm can have serious adverse consequences and it is regarded exclusively as negative behavior within the mental healthcare system, and thus there are many obstacles in relation to being allowed to self-harm. Consequently, several of the young people in our studies have developed different strategies to be allowed to continue self-harm as we will see below.

We are standing at the bus stop, sweating in a crowded group. Once we are inside the bus, way in the back, Lyng tells me about the many different ways they smuggled razor blades inside when they were in the closed ward (which they were for nine months). With their hands, they show me how they glued razorblades to the spectacle frame close to the ear and how they would wear their hair down to hide it further. They elaborate how they would keep razor blades between cleaning wipes, and gently paste razor blades behind cinema tickets and figures in their diary so the razor blades did not fall out even if the book was shaken by the professionals

Another informant, Ida, explains to Anne Mia how it all started with her having an eating disorder (anorexia) and that she was hospitalized when she weighed only 37 kg.

She describes how she lied about symptoms, so she was diagnosed with schizophrenia because she found out that it was “the only thing I could say to keep their mouths shut” (about the eating disorder). Ida explains further: “Otherwise, I had to talk, talk, talk, and they would control all of my food”. But when she started saying that it was the voices in her head, that were telling her to not eat, she finally got some peace to practice her eating disorder. It worked pretty well, as she explains, but unfortunately, one of the side effects from the medicine she was prescribed for schizophrenia was weight gain. So, she would gain weight, and when she did so, she would severely self-harm. She would then be forcibly restrained, and would be put on even more medicine. “Then I self-harmed, then restraint”. She draws a circle in the air with her hand as if to emphasize the repetitive cycle. This pattern continued through several later hospitalizations, where she was admitted after having once again lost a lot of weight.

What these examples with Lyng and Ida point out is that self-harm requires creative strategies developed either through ingenuity or by learning from others, e.g., from social media (see for instance Dyson et al., 2016; Scherr, 2022; Biernesser et al., 2020).

While all our interlocutors stress their necessity for self-harm, the trauma, conflict, illnesses, etc. that underpin this necessity is rarely voiced. It does not mean that it is not experienced, but even if one does not see oneself as being in despair, such as Charlie, a 29-year-old non-binary person diagnosed with bipolar disorder, who during periods of depression burns themselves with a lighter. While visiting Charlie when they were hospitalized owing to increased risk of suicide, they explain how the staff at the ward scolded them (mirroring the experience of Micki) and tried to talk them out of self-harming. Charlie sees themself as “the hero against all the people working against me”. But a Kierkegaardian sense a person can be ignorant of being in despair, and yet still be in despair, for example due to a misrelation of necessity and possibility. Following this line of thought, what Charlie describes might be a sensible act to them, but the self-harm still conveys despair as necessity and possibility are not balanced in their self-relation, other than when turning to self-harm. These strategies are also important focal points, as they emphasize how great the need for self-harm is experienced and can confirm being alive after all, which in many ways is a strong survival mechanism (even if a harmful one). Our data points to the specific and often quite inventive strategies people in our studies make use of to be able to *be* in the world. In this context it is relevant briefly to include Desjarlais’ concept of “struggling along” as a more precise way of describing this way of being in the world. He suggests that “experience is not an existential given but rather a historical possibility predicated on a certain way of being in the world. Since this way of being is only one possibility among many, some people live in terms different from experience” (Desjarlais, 1994, 887). Desjarlais thus proposes a notion of ”struggling along”, which in some cases replaces “experience”. He points out that there are different ways of being in the world framed among other things by temporal orientations: “experience implies a contained, integrative, and occasionally transcendent adaption of sensations, images, and lessons, struggling along entails a diffuse and external rain of distractions that prompts a retreat from the world rather than an incorporation or an assimilation of its parts" (Desjarlais, 1994, 986). The notion of struggling along is a useful analytical tool, because it offers a way of understanding the interlocutors’ contexts for being in the world. For them, to experience the world is often not an accessible position, but on the contrary, something quite diffuse and chaotic. (This difficulty of being in existence is constantly emphasized by Kierkegaard too. See Kierkegaard, 1992, 301, 308, 351). Strategies to be allowed to self-harm and thus feel alive thus become a way to struggle along.

## Enacting Restoration of the Self

None of our informants talk about the pain associated with self-harm, on the contrary: Their body feels numb so burns, cuts, hair-pulling and acid burns are therefore not registered as pain or, at times, even felt at all. But these various forms of self-ham rather convey a way of embodying their bodies. In Kierkegaard’s account of selfhood, making the self is ultimately a task of overcoming despair by working through despair. However, this is a task that human beings can fail, for example through the improper ways of relating to the self or establishing the synthesis, that is, becoming stuck in despair. This is a particular challenge to many of our interlocutors, since despair has become the best known and hence most trusted state they find themselves in. As Billie emphasizes:

“At some point, you are so far out that you only trust in self-harm actions. You do not trust the professionals assigned to help you, mostly because the ones who were supposed to help you have disappointed you throughout life and have taken advantage of your trust. You become suspicious of everybody and everything, so resigning to, for me, cutting, is something I know really well. I know that this will prove that I exist, I know that I can then trust myself. It sounds odd, but being in despair is also being in control”.

We might compare this to Kierkegaard’s descriptions of the way a self must lose itself to become itself (Kierkegaard, 1980). In other words, the self can stand in the way of becoming itself. What we have previously described as the need for performing self-harm is thus a need for being able to access that physical manifestation of despair. Our argument is that self-harm in this sense does not carry exclusively “negative” intentions or connotations for our interlocutors.

Through self-harm subjects explore and seek to restore the self that is on the one hand given, but also find a means to accept the uncertainty of becoming a self when part of the conditions of this coming-into-being is beyond our own making. Overcoming one’s self is an individual task, as Kierkegaard describes: “a relation that relates to itself” (Kierkegaard, 1980, 13).

Self-harm can thus be understood as both dismantling and rebuilding the self. ne objection to this analysis could be, that according to Kierkegaard, we all struggle and find ourselves in despair, as this is part of life. Yet, not all people perform self-harming actions. Here, we need to stress that Kierkegaard’s conception of selfhood emphasizes the individuality of the person, including the individualized existential conditions that mark the person’s lived experience as well as the individual ways in which despair may manifest (or hide) itself. Our interlocutors could then be characterized in a Kierkegaardian sense as living with a form of despair or ‘sickness’ of the self that arises from a misrelation to what it means ‘to be the self that I am’.

For Kierkegaard, such despair is not unique to people who self-harm but is instead a universal human condition. What is distinctive in our interlocutors’ cases is that self-harm becomes their particular way of responding to and negotiating this despair, whereas for others it may manifest in different practices, choices, or modes of being.

## Conclusion

While clinical perspectives often frame self-harm as an injury to the self, our ethnographic material illustrates something more complex. For our interlocutors, self-harm is also a way of mediating overwhelming feelings, struggling along in the midst of instability, and creating a fragile sense of agency within a complex and often chaotic life. These practices are not reducible to pathology; they represent efforts to endure and reconfigure selfhood under conditions of despair and uncertainty.

Our analysis highlights the paradoxical and dialectical nature of self-harm as both destructive and restorative, coercive and emancipatory. Here Kierkegaard’s reflections on despair help illuminate how acts that appear purely negative may also point to or even hold the possibility of self-restoration. His account does not replace but rather complements anthropological approaches that emphasize embodiment and narrative (Lester, 2012; Jenkins, 2010; Csordas & Jenkins, 2018). Using a Kierkegaardian framework sharpens these discussions in seeing despair as a universal human condition: what is distinctive in the case of self-harm is not the presence of despair itself, but that it is worked through the body in violent yet also, paradoxically, life-preserving ways.

At the same time, our interlocutors represent a small, situated group of young people living with mental illness in Denmark. Their accounts cannot be generalized to all experiences of self-harm, and our focus on existential and phenomenological perspectives inevitably leaves out structural and systemic dimensions: Denmark provides universal access to free healthcare, including both inpatient and outpatient psychiatric services. In many other parts of the world, people who self-harm and live with mental illness may face additional challenges such as poverty, limited access to healthcare, and the weight of local or intergenerational traumas. These conditions profoundly shape their existence as well as the ways in which they perceive and engage with self-harm. Nevertheless, by foregrounding lived experience, we hope to have shown how self-harm can be understood not only as symptom or deviance, but also as a practice of negotiating existence and restoring selfhood in fractured, paradoxical ways.

Notes

1. There are different standpoints as to whether eating disorders should be seen as self-harm which we will not dive into here. Instead, we have chosen to include eating disorders, as our interlocutors juxtapose eating disorders with other of their self-harming actions like cutting.

## Data Availability

No datasets were generated or analyzed during the current study.
